# Corporate political activity of the baby food industry: the example of Nestlé in the United States of America

**DOI:** 10.1186/s13006-020-00268-x

**Published:** 2020-04-08

**Authors:** Hacer Tanrikulu, Daniela Neri, Aileen Robertson, Melissa Mialon

**Affiliations:** 1 Department of Nutrition, School of Public Health, University of São Paulo, São Paulo, Brazil; 2 Global Nutrition and Health, University College Copenhagen, Copenhagen, Denmark

**Keywords:** Commercial determinants of health, Food industry, Corporate political activity, Infants and young children feeding

## Abstract

**Background:**

The marketing practices of the breastmilk substitutes industry have been known for decades, but little is known about the influence of the baby food industry, more generally, on public health policy, research and practice, also known as ‘corporate political activity’ (CPA). In this study, the baby food industry refers to for-profit companies that manufacture, market or distribute breastmilk substitutes and food products for infants and young children under two years. In addition, trade associations, public relations firms, marketing agencies and individuals or groups affiliated with the baby food industry are also considered to be part of the baby food industry. The aim of the current study was to systematically identify and monitor the CPA of the baby food industry in the USA, shown by the activities of Nestlé, the largest industry actor in this sector in the country.

**Methods:**

The case study consisted of an analysis of publicly available information for data published between January and November 2018. We included documents from the industry, the government and other sources, including professional organisations, charities and consumer associations. We analysed data using an existing framework to classify the CPA of the food industry.

**Results:**

During the period of data collection, Nestlé employed a list of action-based ‘instrumental strategies’. The most prominent strategy was ‘information strategy’, used to fund, produce and disseminate industry-preferred information. Nestlé was further found to ‘establish relationships with key opinion leaders and health organisations, and the media’, ‘seek involvement in community’ and directly influence policies and programs through indirect access and the placement of actors in government policy settings. The company also used argument-based ‘discursive strategies’ to frame the debate on diet- and public health-related issues.

**Conclusion:**

This study showed that Nestlé used various CPA strategies which may have influenced public health policy, research and practice in ways favourable to the baby food industry. These results could be used to further recognise and pre-empt the influence of corporations on health, in order to ensure that commercial interests do not prevail over public health goals.

## Background

In the USA, early nutrition is compromised in the first two years of life by low rates of exclusive breastfeeding [1], exposure to breastmilk substitutes, early introduction of complementary foods [2] and high consumption of ultra-processed foods [3], practices that go against the World Health Organization (WHO) recommendations [4].

Ultra-processed foods contribute to 58% of all calories consumed by the US pre-school population [3] and there is increasing recognition of the association between dietary patterns based on these products, as defined by the NOVA food classification system [5] and adverse health outcomes [5].

In the USA, among infants born in 2015, only 25% of infants were exclusively breastfed for 6 months, while 17% received breastmilk substitutes within the first 2 days of life, and 28 and 34% before the age of three and 6 months, respectively [1]. In addition to the use of breastmilk substitutes, inadequate and inappropriate complementary feeding practices such as the early introduction of complementary food (before 6 months of age) and frequent consumption of unhealthy foods, to which ultra-processed foods belong, put infants and young children at an increased risk of overweight, obesity and related non-communicable diseases later in life [3].

There is growing evidence that the ‘corporate political activity’ (CPA) of the food industry is a major barrier to the development and implementation of public health policies to promote healthy food environments [6]. The CPA refers to some of the strategies employed by businesses to protect or increase their market shares, by influencing public policy, research and practice [6]. The CPA strategies of the food industry are very similar to those described for the tobacco industry, often referred to as the ‘playbook’, for which we have access to millions of pages of internal documents, after litigation against that industry [6–9]. The CPA of the food industry consists of a list of so-called action-based ‘instrumental strategies’ and argument- based ‘discursive strategies’ [8, 10] (Table 1).

**Table 1:** Description of different corporate political activity strategies of the food industry

Instrumental Strategies	Practices	Mechanisms
Coalition management	Constituency recruitment - Establish relationships with key opinion leaders and health organisations	Promote public-private interactions with health and consumers organisations, among others
		Support professional organisations, including through their funding and / or advertising in their publications
		Establish informal relationships with key opinion leaders
		Support the placement of industry-friendly personnel within health organisations
	Constituency recruitment - Seek involvement in the community	Undertake corporate philanthropy
		Support physical activity initiatives
		Support events (for youth, arts, etc.) and community-level initiatives
	Constituency recruitment - Establish relationships with the media	Establish close relationships with the media, journalists and bloggers, to facilitate media advocacy
	Constituency recruitment - internal	Establish relationships with other actors in the industry
	Constituency fabrication	Establish fake grassroots organisations (‘astroturfing’)
		Procure the support of community and business groups to oppose public health measures
	Opposition fragmentation and destabilisation	Discredit public health advocates personally and publicly
		Infiltrate and monitor the operations and advocacy strategies of public health organisations and advocates
		Create antagonism between health professionals
Information management	Production	Fund research, including through academics, ghost writers, own research institutions and front groups
	Amplification	Cherry pick data that favours the industry, including through the use of non-peer reviewed or unpublished evidence
		Participate in and host scientific events
		Propose industry-sponsored education
	Suppression	Suppress the dissemination of research that does not fit the industry’s interests
		Emphasise disagreement among scientists
		Criticise evidence, and emphasise its complexity and uncertainty
	Credibility	Fronting: conceal industry links to information or evidence, including through the use of scientists serving as advisers, consultants or spokespersons
Direct involvement and influence in policy	Indirect access	Lobby directly and indirectly (through third parties) to influence legislation and regulation so that it is favourable to the industry
		Use the “revolving door”, i.e. ex-food industry staff goes to work in the government, and vice versa
	Incentives	Fund and provide financial incentives to political parties and policy makers (donations, gifts, entertainment or other financial inducements)
	Threats	Threaten to withdraw investments if new public health policies are introduced
	Actor in government decision making	Seek involvement in working groups, technical groups and advisory groups
		Provide technical support and advice to policy-makers
Legal actions	Use legal action (or the threat thereof) against public policies or opponents	Litigate or threaten to litigate against governments, public health professionals and other institutions or individuals
	Influence the development of trade and investment agreements	Influence the development of trade and investment agreements to include clauses favourable to the industry (limited trade restrictions, mechanisms for corporations to sue governments, etc.)
**Discursive strategies**	**Domain**	**Argument**
	The economy	Stress the number of jobs supported and the money generated for the economy
	Governance	Demonise the ‘nanny state’
	Expected food industry costs	Claim that proposed policy will lead to a reduction in sales/jobs
		Claim that cost of compliance will be high for the industry
	Frame the debate on diet- and public health-related issues	Stress the good traits of the food industry
		Shift the blame away from the food industry and its products: focus on individual responsibility, the role of parents, physical inactivity, etc.
		Promote industry’s preferred solutions: education, information, balanced diets, etc.

Legend: Adapted from [8, 10]

‘Instrumental strategies’ aim to achieve industry-preferred policy outcomes and include: ‘coalition management’; ‘information management’; ‘direct involvement and influence in policy’; ‘legal action’ [8, 10]. ‘Discursive strategies’ refer to industry arguments to create the impression that proposed policies may harm public health, the economy or the society [8, 10].

There is growing evidence of the global food industry’s CPA strategies in: Asia [11]; the Western Pacific [12, 13]; Europe [14]; Latin America and the Caribbean [15]. In Brazil, Chile, Ecuador and Fiji, for example, companies in the food industry developed nutrition and physical activity programmes in schools, giving the impression that they were credible experts in these fields, while exposing children to their brands [12, 15]. These programmes also served to shift the blame away from the healthiness of the industry’s products and to the lack of physical activity in the non-communicable diseases epidemic [6, 16]. Lobbying, political donations and other practices that have a direct influence on policy were also regularly used by food industry actors in Australia and Thailand, for example [11, 17].

To date, however, there is limited published evidence of the CPA of the food industry in the infant and young child’s market. The CPA strategies of businesses producing breastmilk substitutes have been known to, and monitored for decades by WHO and UNICEF as well as advocacy groups, such as the International Baby Food Action Network [18, 19]. A recent review by Granheim et al. provided additional supporting evidence on the potential interference of the baby food industry on public health policy through the use of CPA strategies common to the tobacco industry [20].

In 2018, the US delegation caused global outrage for its reported attempts to oppose resolutions promoting breastfeeding at the World Health Assembly and threatening other countries into backing off the resolution, although no clear link with the baby food industry was established [21].

Nestlé is among the leading companies in terms of sales of packaged foods in the USA [22]. In 2014, Nestlé led the US baby food industry by capturing 34% of the market [23]. Nestlé’s political influence such as lobbying the US government and agencies has been documented by civil society organisations [24], but there is currently little published evidence and research on this topic.

The aim of the current study was to systematically identify and monitor the CPA of the baby food industry in the USA for the period January to November 2018, shown by the activities of Nestlé, the largest industry actor in this sector in the country. Our intention was to emphasise the necessity to investigate and document the day-to-day CPA of that industry, rather that its influence on a specific policy or event.

## Methods

This study employed a step-by-step systematic approach recommended by Mialon et al. [6].

### Selection of food industry actors

In this study, the baby food industry refers to for-profit companies that manufacture, market or distribute breastmilk substitutes and food products for infants and young children under 2 years. In addition, trade associations, public relations firms, marketing agencies and individuals or groups affiliated with the baby food industry are also considered to be part of the baby food industry.

We conducted a pilot study with data from Nestlé as the most prominent actor in the baby food industry. We were able to locate a large amount of data for the company. This led our decision to only select this industry actor for our study.

For this study, we included the following actors from the company:

- its subsidiary Gerber;
- Gerber BabyNes;
- the Nestlé Nutrition Institute;
- the Nestlé-owned Wyeth Nutrition Science Center;
- the global operator Nestlé SA activities that were US-based.

### Identification of sources of information

We collected publicly available information on the internet. Sources included industry’s own materials (from its websites, Twitter accounts); government materials (registers for lobbyists or websites of agencies responsible for infants and young children nutrition and health-related issues); and websites of: universities; professional organisations; charities; civil society organisations; and consumer associations [6]. We also conducted searches on Google News. In total, we searched more than 60 websites, as described in Additional file 1.

### Data collection and analysis

HT led the data collection and analysis. We collected data that was published between January and November 2018. We included the most recent data from annual or occasional events and publications, including the latest annual reports from Nestlé [25].

We collected data over a 3-month period, between August–November 2018. We collected information from the BabyNes webpages after the completion of data collection as they had been missed during that period.

We undertook a thematic qualitative analysis in an iterative process. Data collection involved reading all sources of information followed by identifying and categorizing CPA practices according to the adapted Policy Dystopia Model [8, 10]; data were collected in an Excel spreadsheet. All data are available as Additional file 2. Each piece of data is allocated with a unique code starting with the letter A followed by a number (e.g. A1).

DN reviewed 100% of all codes, including their categorization in themes. MM and AR reviewed a random sample of 10% of all the codes. Mutual agreement was reached between the four researchers, through discussions, for the final categorization of CPA practices.

Adapted from the tobacco industry, the CPA practice ‘constituency recruitment- internal’, already identified in the literature for the tobacco industry [8], was added within the category ‘coalition management’. This practice enables the establishment of alliances among with organisations and associations whose founding members are representatives of corporations that recruit their employees to carry out action on their behalf [8, 10].

Results are presented through a narrative synthesis with illustrative examples of the most prominent CPA strategies employed by Nestlé in the USA during the period of analysis. The use of direct quotations of collected data is employed to illustrate examples in the results section.

## Results

Table 2 gives an overview of the different CPA strategies and practices used by Nestlé for the period of analysis. We documented four hundred and thirty-eight examples of CPA practices. Nestlé, for the period of analysis, used all ‘instrumental strategies’ previously identified in the literature, with the exception of the ‘legal action’ strategy. We also found evidence that Nestlé employed argument-based discursive strategies (we collected sixty-three examples) which could influence public opinion. ‘Information management’ was documented as the most prominent CPA strategy, followed by: ‘coalition management’; ‘discursive strategies’; and ‘direct involvement and influence in policy’.

**Table 2:** Occurrences of CPA strategies and practices used by Nestlé during the period January to November 2018

CPA strategies and practices	Total occurrences
**Coalition management**	69
Establish relationships with key opinion leaders and health organisations	44
Seek involvement in the community	20
Establish relationships with the media	3
Constituency fabrication	0
Constituency recruitment – internal	2
Opposition fragmentation and destabilization	0
**Information management**	281
Production	48
Amplification	231
Suppression	0
Credibility	2
**Direct involvement and influence in policy**	25
Indirect access	17
Incentives	0
Threats	0
Actor in government decision making	8
**Legal actions**	0
Use legal action against public policies or opponents	0
Influence the development of trade and investment agreements	0
**Discursive strategies**	63
The economy	1
Governance	0
Expected food industry costs	0
Frame the debate on diet- and public health related issues	62
**Total occurrences**	**438**

### Information management

Two hundred eighty-one examples were categorized within the strategy ‘information management’.

#### Production

The practice of ‘production’ refers to industry-funded research including through academics, ghost writers, own research institutions and front groups and is used to produce industry-favorable information [8, 10]. In this study, we collected 48 examples under this CPA practice.

The Nestlé Research Center in Switzerland conducted the ‘Nestlé Feeding Infants and Toddlers Study (FITS)’ (A403), which is described as the largest dietary intake study in the USA to examine the eating habits of infants and children under the age of four (A404, A413, A420, A436–7). Advisers were a “team of pediatric experts and nutrition scientists from leading academic, medical, government and research institutions” (A404), and authors of the published data included employees at the Nestlé Research Center and Nestlé Nutrition/Gerber Products Company (A429, A421–435). Nearly 10,000 parents and caregivers were surveyed across three FITS surveys and the results of the project were published in more than 50 peer-reviewed scientific articles published over fifteen years (A395).

#### Amplification

The practice of ‘amplification’, with 231 documented examples, was applied through different mechanisms including: selecting industry-preferred evidence; disseminating industry-sponsored and preferred information and evidence; providing industry-sponsored education; and participating in and hosting scientific events.

As a self-described “leader in early childhood nutrition” (A219), Nestlé emphasized that “research informs everything (we) do” (A219) at Nestlé US subsidiary Gerber “from the products we make, the nutrition education we deliver and the services we offer” (A219). Nestlé repeatedly drew on data from the FITS study (A203, A251) to “provide resources (…) for health care professionals at Medical.Gerber.com and for parents at Gerber.com” (A219).

Nestlé used key FITS findings to provide dietary recommendations and tips for parents (A163, A229, A231, A278). FITS findings were further used to disseminate information through education programs for parents (A203) and scientific events (A284, A350). Gerber developed new products in response to the results of the study. Then, key finding from FITS were in turn used to highlight nutritional shortcomings, such as lack of micronutrients and unhealthy eating behaviour, among infants and young children and promote these new products to parents (A243, A251, A271, A276, A278).

FITS findings were used for references in the handbook for Pediatric Nutrition published by the American Academy of Pediatrics (AAP) to highlight low rates of children consuming iron-fortified cereals (A387) and low fat intake among young children (A388). Nestlé referred to the AAP also as a source for the nutritional value of their products (A180) and to emphasise that Nestlé resources were consistent with AAP nutritional guidelines (A181). Nestlé regularly stated that the latest FITS research findings were “well-timed to inform food policy discussions, including the development of the Dietary Guidelines for Americans 2020-2025 and reconsideration of the benefits offered in the WIC food package which was last revised in 2009” (A349).

Nestlé’s website for healthcare professionals is apparently a platform where paediatric health care professionals can access information, resources and tools to support their daily practice and share with their patients (A181, A220). Such resources included: menu planners (A181, A221) and nutritional guidelines for infants and young children (A181).

Nutritional guidelines according to this system were given depending on infants and young children’s “oral motor development capability” (A227) where for example, infants older than 4 months of age, also referred to as the “supported sitter” stage (A230), were stated to be ready to be introduced to solid foods, such as infant cereals, vegetables and fruits (A223, A227, A230, A232, A306-A308).

Nestlé further provided WHO growth charts to medical professionals (A211, A385), as resources to support parents track the development of their infants (A211). On the chart, Nestlé placed their own Gerber logo next to the US’s Centers for Disease Control and Prevention logo (A385). This creates the impression of potential endorsement by health organisations.

The company also disseminated information through its online support center called “Gerber Start Healthy, Stay Healthy Resource Center” (A224), where a Nestlé-backed team of nutrition experts, including Registered Dietitians (A224, A235, A380), International Board Certified Lactation Consultants (A170, A224) and Breastfeeding Educators/Certified Lactation Educators (A235), provide information about infant and young child nutrition. Nestlé’s Registered Dietitians were “available for questions on prenatal nutrition and nutrition for infants and young children 0–48 months” (A224), while Lactation Educators were “available for live counselling on a one-to-one basis” (A224) to “(d)iscuss any breastfeeding concerns” that mothers may have (A170).

#### Credibility

Credibility is defined as a CPA practice that attempts to conceal the links or support that an industry has to the creation of information, such as enlisting scientists who are paid to be advisers or spokespersons on infant health and nutrition [10]. In a press release published on the company’s website, a researcher expressed her support for the “need for dietary guidance (that) starts very early at birth or even earlier” (A390) and welcomed the FITS findings to help create such guidance. However, this researcher’s responsibilities as a co-author and coordinator of the FITS supplement (A423) were not transparent. Similarly, researchers’ affiliation to FITS were not disclosed in the list of speakers for a Nestlé-sponsored event called “A Healthy Start: The Infant and Early Childhood Nutrition” (A389) where key health officials, including congressional members, lawmakers and experts came together to discuss policies on early childhood nutrition.

### Coalition management

We classified sixty-nine examples of CPA as ‘coalition management’.

#### Establish relationships with key opinion leaders and health organisations

Nestlé established relationships with key opinion leaders, health organisations and universities by promoting public-private interactions. For example, as a member of the AAP’s “Corporate Friends of Children Fund” (A57), Nestlé provided over US$50,000 to enable the AAP to “continually generate new knowledge about the best way to care for children” (A57). In return, donors “receive significant acknowledgement in several AAP publications visible to (…) 60,000 members” (A57). This might explain why Nestlé was acknowledged in an online version of AAP’s handbook for Pediatric Nutrition (2013) where Nestlé provided a grant to render an online version of the handbook to be accessible for the general public free-of-charge (A40). Moreover, three members of the Gerber Advisory Council held important positions at the AAP including: being members of the AAP’s Committee on Nutrition (A33, A34); and being an associate editor of the AAP handbook (A35). A former chair of the AAP’s Committee on Nutrition (2012–2013) was further involved in the preparation of the AAP handbook (A33) [26], while also serving both on the Editorial Board of a paediatric journal, ‘Annales Nestlé’ and on the Executive Committee of the Nestlé Nutrition Institute (see the declarations in [27]). In addition, Nestlé appeared to be one of the founding sponsors of the AAP’s Institute for Healthy Childhood Weight (A28-A31, A38, A41), which focuses on paediatric obesity prevention by providing funds to successful applications submitted to the Institute (A29). One example of funded program is “Healthy Active Living for Families” which is designed to prevent early childhood obesity (A41-A44, A58). Table 3 lists examples of professional organisations, events and conferences that Nestlé supported.

**Table 3:** List of events/programs Nestlé supported for the period January to November 2018

Name of event/program	Dates	Role of Nestlé
2018 National Child Nutrition Conference (A65)	April 19–21, 2018	Sponsor
AAP experience. National Conference & Exhibition (A59)	November 2–6, 2018	Exhibitor
NASPGHAN (North American Society for Pediatric Gastroenterology, Hepatology and Nutrition) Foundation/ Nestlé Nutrition Research Young Investigator Development Award (A45)	Submission deadline July 6, 2018	Award funder and foundation partner (A46)
American Society Nutrition 2018 - Samuel J. Fomon Award for Young Investigators (A27)	June 19, 2018	Funder and sustaining partner (A48)
Flux Satellite Conference by University of Chapel Hill (A53)	May 6–8, 2018	Sponsor
Continuing medical education on 12th Advances in Pediatric Nutrition by John Hopkins Medicine (A66)	August 4, 2017- August 3, 2019	Grant provider
Event “A Healthy Start: The Infant and Early Childhood Nutrition”, attended by nutrition experts and lawmakers (A67)	September 12, 2018	Sponsor

#### Seek involvement in local communities

The CPA practice ‘seek involvement in local communities’ refers to activities such as undertaking corporate philanthropy or supporting events and community-level initiatives [8, 10]. In this study, Nestlé supported local community initiatives: in the State of New Jersey, for example, Nestlé in partnership with Rutgers University-Newark and the New Jersey YMCA State Alliance, supported “Start Healthy, Stay Healthy”, an early nutrition education program implemented in five different locations across the country (A1, A3, A5, A7-A11, A13-A15, A19–20).

“Gerber continues the Early Childhood Nutrition Education program in an effort to tackle childhood obesity rates in local communities by teaching parents and caregivers of young children simple ways to improve the diets of infants, toddlers, and pre-schoolers. The program covers topics including the importance of breastfeeding, hunger and fullness cues, introduction to solids, transitioning to table foods, healthy snacking, and feeding the fussy toddler.” (A3).

Another initiative Nestlé supported was the 28th National Baby Food Festival in Fremont, Michigan, where the Nestlé Gerber headquarters is located (A2, A6). As part of the festival, adults enrolled in ‘baby food eating contest’, wearing Gerber T-shirts and tasting Gerber baby food products.

#### Establish relationship with media

Nestlé established relationships with the media by facilitating media advocacy: while one Nestlé representative discussed FITS findings on TV (A68), another presented in an interview, published in a newspaper, an initiative where parents were supported with an educational program including infants and young children feeding (A69).

#### Constituency recruitment - internal

Two examples have been documented in this research and show Nestlé’s use of ‘internal constituency recruitment’ from within the baby food industry. Nestlé was part of two industry-led umbrella organisations, namely the Infant Nutrition Council “an association of manufacturers of infant formulas, follow-up formulas or growing up milks” (A21) with members including Abbott Nutrition, Perrigo Nutritionals and Reckitt Benckiser (A21) and a new trade association called Sustainable Food Policy Alliance, which Nestlé launched along with the U.S. divisions of Danone, Mars and Unilever in 2018 (A22).

### Direct involvement and influence in policy

We classified twenty-five examples of CPA as ‘direct involvement and influence in policy’. For example, it was noted that businesses tend to “work with the U.S. Department of Agriculture and Health and Human Services on helping to shape science-based recommendations for encouraging healthy diets at all life stages” (A93), especially when Dietary Guidelines for Americans are being developed. Also as a member of the “National Strategic Partners through the USDA (United States Department of Agriculture) Center for Nutrition Policy and Promotion’s Nutrition Communicators Network”, Gerber was involved with the development of “MyPlate”, the current food guide icon used to share information from the dietary guidelines, and “(t)hrough this partnership, Nestlé brands (including Gerber) share nutrition information to promote the Dietary Guidelines for Americans” (A89).

### Discursive strategies

During data collection, we identified sixty-three examples of discursive strategies, where Nestlé used different arguments that would be beneficial to its company and activities. One example of CPA appeared to emphasize the company’s role in supporting the economy by creating new jobs (A87). We found sixty-two examples under ‘frame the debate on diet- and public health-related issues’. Nestlé showed its support for physical activity to prevent obesity during infancy (A95–6). The company also emphasised its support for breastfeeding practices (A150, A153,) and the WHO Code of Marketing of Breastmilk Substitutes (the WHO Code) (A116, A157), and said that it never lobbied governments, including the US government, to undermine breastfeeding policies (A144).

### A case study of WIC and the CPA of Nestlé

In 2018, in almost all US states, Nestlé was the company contracted by government programs (A71) [28, 29]. Nestlé’s products were used by Medicaid and the Special Supplemental Nutrition Program for Women, Infants, and Children (WIC), which provides, among others, infant formula (designated for infants 0–12 months) for low-income families at no cost. WIC can serve as an illustration of how the company combines different CPA strategies in the USA.

**Fig. 1:**
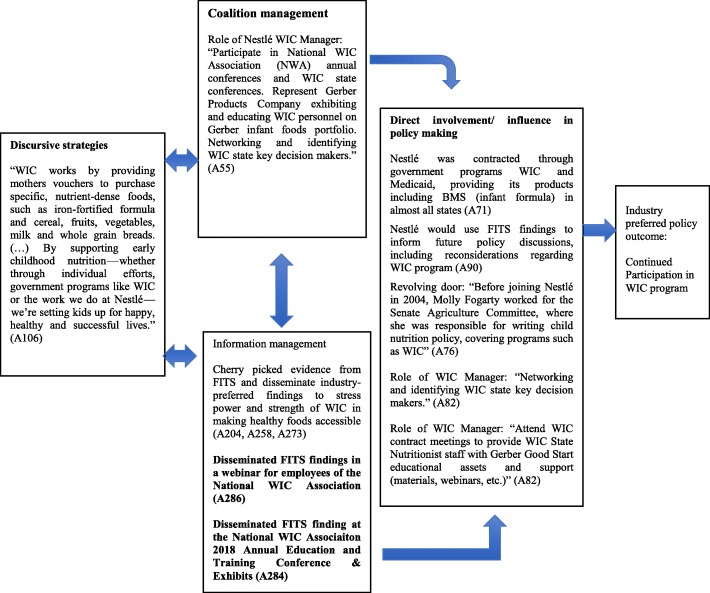
‘Policy Dystopia Model’ adapted to the baby food industry in the USA. Footnote: Adapted from Ulucanlar S, Fooks GJ & Gilmore AB. The Policy Dystopia Model: An Interpretive Analysis of Tobacco Industry Political Activity. PLoS Med. 2016; 13, 1–21

Infant formula, a breastmilk substitute designated for infants aged 0–12 months, is profitable in the USA, for two reasons: cow’s milk, the main ingredient,; is cheap due to federal subsidies [30]; and government purchases for the WIC program account for more than half of all infant formula sold in the country [29]. WIC procures the designated breastmilk substitutes through a bidding process, in which manufacturers like Nestlé offer discounts in the form of large rebates, so that WIC ends up paying only about 8% of the wholesale price [29]. Despite large discounts, it has been shown that winning a contract with WIC increased breastmilk substitutes manufacturers’ market share by 74%, mostly due to WIC recipients switching to the newly contracted brand and a spill-over effect, in which sales of breastmilk substitutes purchased outside WIC increased [29]. Considering the profitability of the WIC program, it comes with no surprise that Nestlé employed various CPA strategies, appearing to support and stress the importance of the continuation of this program (Fig. 1).

In the past, Nestlé’s interest in the WIC program was shown through its lobbying activities. The Center for Responsive Politics documented that the company spent US$160,000 lobbying the US government on issues related to the WIC program in 2014 [31]. Our study findings suggest that Nestlé’s participation and efforts to support the WIC programme create a conflict of interest, where commercial interests may undermine optimal breastfeeding practices.

We also noted that the company intended to employ its own WIC manager to carry out “(n)etworking and identifying WIC state key decision makers.” as well as attending “WIC contract meetings to provide WIC State Nutritionist staff with Gerber Good Start educational assets and support (materials, webinars, etc.)” (A82).

In addition, the latest Nestlé-led data showed that WIC is effective is enabling low-income parents to purchase specific foods such as breastmilk substitutes, including infant formula (A106).

“WIC works by providing mothers vouchers to purchase specific, nutrient-dense foods, such as iron-fortified formula and cereal, fruits, vegetables, milk and whole grain breads. The latest data shows the program is helping small children get the nutrients their growing bodies need during the most critical stages of development. (…) WIC vouchers make it possible, and easy, for parents to give their children these important nutrients. By supporting early childhood nutrition - whether through individual efforts, government programs like WIC or the work we do at Nestlé - we’re setting kids up for happy, healthy and successful lives.” (A106).

## Discussion

In this study, we documented a total of 438 examples of CPA practices used over a period of 10 months, during 2018. We found that ‘discursive strategies’ were mostly used to ‘frame the debate on diet- and public health-related issues’, and these were often used in combination with ‘instrumental strategies’, as illustrated in our case study of the WIC program.

‘Discursive strategies’ stressed the industry ´s good intentions (education programs); focused on the role of parents; and promoted industry preferred solutions (education, voluntary instead of mandatory regulatory measures). For example, public health efforts to prevent early childhood obesity were provided through offering parental education and recommending voluntary marketing regulations for food and beverages. Voluntarily offering assistance to be part of the solution and not part of the problem [6] appears to help the industry gain goodwill and so have more potential to shape public opinion and persuade policy makers to influence policies, programs or regulations towards industry interests [8].

The most frequent instrumental CPA documented in this study where those strategies classified under ‘information management’. Information on infant and young children nutrition and health, created by the industry, was targeted both at the general public, including mothers and caregivers, and health professionals. Indeed, Nestlé employed nutrition and health professionals whose role is to provide information to parents and health professionals free of charge. Concerns have frequently been raised about these kinds of activities and their potential conflict of interest [18, 32, 33]. Other examples exist where Nestlé attempted to frame the debate around the WHO Code. Nestlé’s industry-led breastmilk substitutes policy differed from the Code in several aspects, potentially creating opportunities for continued promotion of breastmilk substitutes [18]. Nestlé built coalitions with key opinion leaders such as: health professionals; researchers; civil society organisations; and local communities. Such alliances seem to help to create platforms that strengthen a company’s credibility and provide a forum to present arguments and take actions to influence public policies and programs. Some paediatricians who were members of AAP committees also had ties with the companies. This may have influenced the AAP’s dietary guidelines for infants and young children. At the very least an infant food company is likely to gain respect and credibility from the general public and goodwill from health professionals where collaboration is established. In addition, if these services are not available through the public health care system it allows opportunities for actors of the infant food industry to directly contact the public, which is a violation of the WHO Code [18]. This Code recognises that many “health workers, women, and families are susceptible to direct and indirect marketing strategies” [34]. Nestlé’s membership of the Sustainable Food Policy Alliance raises questions about the potential conflict of interest and ability to lobby the US government policy, including nutrition (A71). The use of legal action was not found in this study.

This study has a number of limitations. We only collected information from the public domain. This could have led to an incomplete analysis of the entire range of CPA strategies used within the infants and young children food sector. Moreover, our results are based on this information, and, perhaps, if more data were available, different conclusions might have been made. Practices such as lobbying directly towards policy makers through private interactions are not usually transparent and not readily available in the public domain [6]. Other similar studies carried out interviews with key informants to obtain additional information [12, 17]. Another major limitation is that this study attempts to document the CPA strategies used by only one infant food industry. Limiting the study to only one actor may result in the findings not being generalizable and representative of all the CPA strategies being used within the infants and young children food sector. However, this allowed an in-depth investigation to be carried out. In addition, the data represents only a snapshot of CPA practices that were carried out during a 10-month period and does not illustrate whether or not there are different trends over time. Future investigations could include: a larger range of different industry actors; conducting interviews with key opinion leaders; and collect data looking at trends over time. Moreover, this research documents a range of CPA strategies and cannot provide direct evidence of intent to influence [6]. Yet the most likely incentive for the use of CPA strategies is similar to those of marketing which is to the increase market share and protect the interests of shareholders [35]. There is a risk that such practices may be translated into political power to protect companies’ profits at the potential expense of public health [6, 22]. For example, Nestlé’s CPA may help: undermine optimum levels of breastfeeding; prevent the adoption and implementation of the WHO Code of Marketing of Breastmilk Substitutes; delay the cessation of the availability of free infant formula within the WIC program; and undermine the development of optimal US dietary guidelines for infants and young children.

The CPA strategies described here are similar to strategies previously documented as used by other industries [10, 12, 15, 18, 30, 36, 37] including the baby food industry [20], the alcohol and tobacco industries [6, 8, 22]. We have used an existing framework for classifying the CPA of the tobacco industry [8], adapted to the food industry [10] and an existing approach to data collection to identify and monitor the CPA of the food industry [10]. Given the similarity between the strategies used by different industries, the findings of the present study, lend strength and validity to the used methods. Therefore, this study can be expected to be replicated using the same systematic approach in order to study the CPA of the baby food industry in other countries.

While there is a large literature on violations of the WHO Code, there is limited research that documented the corporate influence of the baby food industry at the country level. Hence, more research and continued monitoring of the CPA of prominent actors of the baby food industry could help to persuade policy makers to develop regulatory and policy measures to challenge and bring an end to the interference of the baby food industry in public policies and programs. At a minimum, such measures should include efforts to improve transparency and accountability for any private-public interactions between governments, health professional associations, civil society organisations and the private sector such as the food industry.

## Conclusion

This study showed that Nestlé used various CPA strategies which may have influenced public policy, research and practice in ways favourable to the industry. The findings of this study could be used by public health advocates, civil society originations, the media and the public to further recognise and pre-empt the influence of corporations on health, in order to ensure that commercial interests do not prevail over public health goals.

## Supplementary information

**Additional file 1.** Sources of information for the study of the CPA of Nestlé in the USA. List of sources consulted for data collection.

**Additional file 2.** All data collected for the study of the CPA of Nestlé in the USA. All raw data and associated relevant information collected for the study.

## Data Availability

All data generated or analysed during this study are included in this published article (and its additional files).
