# Supplementary material for: Corporate political activity of the baby food industry: the example of Nestlé in the United States of America
Source: Int Breastfeed J. 2020 Apr 8;15:22. doi: 10.1186/s13006-020-00268-x (PMC7140353; doi:10.1186/s13006-020-00268-x)
Supplement: Supplementary file 1 — Additional file 1. Sources of information for the study of the CPA of Nestlé in the USA. List of sources consulted for data collection. [file 13006_2020_268_MOESM1_ESM.docx]

**Additional file 1:** Sources of information for the study of the CPA of Nestlé in the USA^[[1]](#footnote-1)^

| Nature of the source of information | Category | Source of information |
| --- | --- | --- |
| Food industry material | Country-specific website of the industry actor | **Nestle Gerber and Nestlé USA:**  https://www.gerber.com/Home  http://news.gerber.com/  https://medical.gerber.com/  https://www.nestlenutrition-institute.org/  https://www.nestlehealthscience.com/  https://www.nestleusa.com/  https://www.nestle.com  https://www.babynes.com/us-en  https://twitter.com/NestleUSA |
| Government material: Departments (and related agencies) responsible for diet- related issues | Websites of departments and related agencies in charge of health and infant and young child nutrition (National level) | **US Department for Agriculture (USDA):** https://www.usda.gov/  **USDA Food and Nutrition Service**: <https://www.fns.usda.gov/>  **USDA Center for Nutrition Policy and Promotion (CNPP):** https://www.cnpp.usda.gov/  **USDA Dietary Guidelines:** <https://www.dietaryguidelines.gov/>  **USDA Nutrition:** <https://www.nutrition.gov/>  **USDA Food Nutrition Information Center:** <https://www.nal.usda.gov/fnic>  **USDA Choose MyPlate.gov**: https://www.choosemyplate.gov/  **USDA National Institute of Food and Agriculture**: <https://nifa.usda.gov/topic/nutrition>  **US Human and Health Services:** <https://www.hhs.gov/>  **Food and Drug Administration (FDA) Infant Formula Guidance Documents and Regulatory Information**: <https://www.fda.gov/Food/GuidanceRegulation/GuidanceDocumentsRegulatoryInformation/InfantFormula/default.htm>  **Administration for Children and Families:** <https://www.acf.hhs.gov/about>  **Office of Disease Prevention and Health Promotion(ODPHP):**  https://health.gov/  https://healthfinder.gov/  https://www.healthypeople.gov/  **National Institutes of Health**: <https://www.nih.gov/>  **Center for Disease Control and Prevention (CDC**): https://www.cdc.gov/  **National WIC Association**: https://www.nwica.org/ |
| Other materials | Universities with a School/ Department of nutrition/dietetics/exercise or physical activity | **Augusta University – Medical College of Georgia:** https://www.augusta.edu/mcg/  **Indiana University School of Medicine**: https://medicine.iu.edu/  **John Hopkins Bloomberg School of Public Health**: https://www.jhsph.edu/  **Ohio State University**: https://www.osu.edu/  **Rutgers University‒Newark**: https://www.newark.rutgers.edu/  **University of California, Davis**: https://www.ucdavis.edu/  **University of California, San Diego**: https://ucsd.edu/  **University of Connecticut – Rudd Center for Food Policy and Obesity**: http://www.uconnruddcenter.org/  **University of North Carolina - Chapel Hill**: https://www.unc.edu/  **US Department of Agriculture (USDA) Nutrient Data Laboratory: Beltsville, MD:**  <https://www.ars.usda.gov/northeast-area/beltsville-md-bhnrc/beltsville-human-nutrition-research-center/nutrient-data-laboratory/> |
|  | Industry-led trade and consumer associations and other organizations | **Grocery Manufacturers Association:** https://www.gmaonline.org/  **Facts Up Front:** http://www.factsupfront.org/ Done  **Sustainable Food Policy Alliance:** <https://foodpolicyalliance.org/>  **International Food Information Council Foundation: h**ttps://www.foodinsight.org/about  **Food Marketing Institute:** <https://www.fmi.org/about-us>  **Infant Nutrition Council:** https://infantnutrition.org/about-inca/ |
|  | Professional Organisations, charities | **American Academy of Pediatrics (AAP)**:  https://www.aap.org/en-us/Pages/Default.aspx  AAP healthychildren.org  AAP publications: http://www.aappublications.org/  **Academy of Nutrition and Dietetics:** https://www.eatright.org/  **Academy of Nutrition and Dietetics Foundation:** https://eatrightfoundation.org/  **Food Research & Action Center (FRAC):** <http://www.frac.org/>  **American Obesity Association**: <https://www.obesity.org/>  **Association of American Medical Colleges**: <https://www.aamc.org/>  **American Public Health Association**: <https://www.apha.org/>  **North American Society for Pediatric Gastroenterology, Hepatology and Nutrition**: <https://www.naspghan.org/>  **Fed is Best Foundation:** https://fedisbest.org/  **International Lactation Consultant Association**: <https://www.ilca.org/why-ibclc/falc>  **US Lactation Consultant Association**: <https://uslca.org/> |
|  | Major conferences on diet-, public health- or physical activity-related issues (National level) | **AAP Conference:** https://aapexperience.org/  **Obesity Week (conference):** https://obesityweek.com/  **Academy of Nutrition and Dietetics- Food and Nutrition Conference and Expo**: https://eatrightfnce.org/  **American Society for Nutrition meetings**: https://meeting.nutrition.org/ |
| Media | News and media releases | - Search on Google News with the name of the company and “health”  Search on company name and name of selected online news sources for the period 1 January - 1 Nov 2018  -New York Times  -The Wall Street Journal  -USA Today  -Huffington Post  -The Hill |
| Other | Open Secrets – information on lobbying | https://www.opensecrets.org/lobby/clientsum.php?id=D000042332&year=2018 |

1. Adapted from Mialon M, Swinburn B & Sacks G. A proposed approach to systematically identify and monitor the corporate political activity of the food industry with respect to public health using publicly available information. Obes. Rev. 2015; 16: 519–530. [↑](#footnote-ref-1)
